# Maternal prenatal depression is associated with decreased placental expression of the imprinted gene *PEG3*

**DOI:** 10.1017/S0033291716001598

**Published:** 2016-08-15

**Authors:** A. B. Janssen, L. E. Capron, K. O'Donnell, S. J. Tunster, P. G. Ramchandani, A. E. P. Heazell, V. Glover, R. M. John

**Affiliations:** 1 Cardiff School of Biosciences, Cardiff University, Cardiff CF10 3AX, UK; 2 Centre for Mental Health, Imperial College, Hammersmith Campus, London W12 0NN, UK; 3 Douglas Mental Health University Institute, 6875 La Salle Boulevard, Verdun, Quebec H4H 1R3, Canada; 4 Maternal and Fetal Health Research Centre, University of Manchester, Manchester, UK; 5 Institute of Reproductive and Developmental Biology, Imperial College London, Hammersmith Campus, Du Cane Road, London W12 0NN, UK

**Keywords:** Human placental lactogen, *PEG3*, prenatal depression

## Abstract

**Background:**

Maternal prenatal stress during pregnancy is associated with fetal growth restriction and adverse neurodevelopmental outcomes, which may be mediated by impaired placental function. Imprinted genes control fetal growth, placental development, adult behaviour (including maternal behaviour) and placental lactogen production. This study examined whether maternal prenatal depression was associated with aberrant placental expression of the imprinted genes paternally expressed gene 3 (*PEG3*), paternally expressed gene 10 (*PEG10*), pleckstrin homology-like domain family a member 2 (*PHLDA2*) and cyclin-dependent kinase inhibitor 1C (*CDKN1C*), and resulting impaired placental human placental lactogen (*hPL*) expression.

**Method:**

A diagnosis of depression during pregnancy was recorded from Manchester cohort participants’ medical notes (*n* = 75). Queen Charlotte's (*n* = 40) and My Baby and Me study (MBAM) (*n* = 81) cohort participants completed the Edinburgh Postnatal Depression Scale self-rating psychometric questionnaire. Villous trophoblast tissue samples were analysed for gene expression.

**Results:**

In a pilot study, diagnosed depression during pregnancy was associated with a significant reduction in placental *PEG3* expression (41%, *p* = 0.02). In two further independent cohorts, the Queen Charlotte's and MBAM cohorts, placental *PEG3* expression was also inversely associated with maternal depression scores, an association that was significant in male but not female placentas. Finally, *hPL* expression was significantly decreased in women with clinically diagnosed depression (44%, *p* < 0.05) and in those with high depression scores (31% and 21%, respectively).

**Conclusions:**

This study provides the first evidence that maternal prenatal depression is associated with changes in the placental expression of *PEG3*, co-incident with decreased expression of *hPL*. This aberrant placental gene expression could provide a possible mechanistic explanation for the co-occurrence of maternal depression, fetal growth restriction, impaired maternal behaviour and poorer offspring outcomes.

## Introduction

Maternal psychological stress (or prenatal stress) during pregnancy, such as symptoms of anxiety and/or depression, has been associated with fetal programming of adverse long-term consequences for the child including an increased risk of fetal growth restriction (for example, Steer *et al.* 1992; Rondo *et al.* 2003; Khashan *et al.* 2008; Liu *et al.* 2012), emotional and behavioural problems, learning difficulties, cognitive impairment and psychopathology (for reviews, see Van den Bergh *et al.* 2005; Talge *et al.* 2007). In addition, there is evidence that prenatal depression can impair the normal development of postnatal maternal behaviour and mother–infant interactions (Pearson *et al.* 2012).

Located at the boundary between the maternal and fetal environments, the placenta supports fetal growth and development through numerous functions including transport of nutrients and oxygen and the production and metabolism of hormones (Gude *et al.* 2004). The placenta has been proposed as a potential mechanism mediating the association between maternal prenatal stress and adverse infant outcomes (O'Donnell *et al.* 2009; Janssen *et al.* 2016). Consistent with this hypothesis, prenatal stress has previously been associated with altered placental function in both animal models (Mairesse *et al.* 2007; Jensen Pena *et al.* 2012) and in humans (O'Donnell *et al.* 2012; Blakeley *et al.* 2013; Reynolds *et al.* 2015).

Imprinted genes, which are monoallelically expressed with expression depending on the parent of origin (Surani, 1998), are regulated by epigenetic marks that may respond to *in utero* environmental stimuli. These genes have been suggested as potential mediators of adverse infant outcomes because of their well-established roles in controlling fetal growth, placental development, adult behaviour and metabolism (Lefebvre *et al.* 1998; Li *et al.* 1999; Curley *et al.* 2005; Smith *et al.* 2006; Tunster *et al.* 2013; Jensen *et al.* 2014; McNamara & Isles, 2014). Aberrant expression of imprinted genes in the placenta has also been demonstrated to be associated with impaired infant neurobehavioural development in humans (Green *et al.* 2015). Recent studies demonstrate that a subset of imprinted genes converge on the endocrine lineages of the mouse placenta to regulate placental hormone production (John, 2013). This may be of particular relevance to maternal prenatal stress as the placenta is a significant source of hormones, such as placental lactogens (Glynn & Sandman, 2011). These lactogenic hormones act on the maternal brain, priming the mother for pregnancy and postnatal care (Bridges *et al.* 1985, 1990, 1997; Bridges & Freemark, 1995; Glynn & Sandman, 2011). In addition, impaired placental lactogen production has been associated with adverse infant outcomes such as fetal growth restriction (Roh *et al.* 2005; Dutton *et al.* 2012). Thus, it is possible that aberrant placental imprinted gene expression and resulting impaired placental lactogen production mediate the association between maternal prenatal stress and adverse infant outcomes. In support of such a hypothesis, epigenetic changes in cord blood DNA at imprinted loci have been associated with both depressed maternal mood (Liu *et al.* 2012) and maternal stress (Vidal *et al.* 2014). The expression of imprinted genes in the placenta has not previously been examined in relation to maternal prenatal stress.

In this study we examined the expression levels of the paternally expressed gene 3 (*PEG3*), paternally expressed gene 10 (*PEG10*), pleckstrin homology-like domain family a member 2 (*PHLDA2*) and cyclin-dependent kinase inhibitor 1C (*CDKN1C*). These four imprinted genes were chosen based on the conserved imprinting status between mouse and human and their predicted role in regulating the production of placental hormones, including the placental lactogens, known to induce physiological changes in pregnant women (John, 2013). Expression of these genes was first analysed in a pilot cohort of women (the Manchester cohort) with clinically diagnosed depression during pregnancy, with results suggesting a significant alteration in placental *PEG3*. Based on these results, we further analysed placental *PEG3* expression in two additional independent cohorts of mothers reporting prenatal symptoms of depression. Finally, given the proposed link between *PEG3* expression and placental hormones, we quantified expression of human placental lactogen (*hPL*, also known as chorionic somatomammotropins; CSH) in all three cohorts.

## Method

### Manchester cohort

Placental gene expression was first analysed in a pilot cohort. Women (*n* = 75) presenting with maternal perception of reduced fetal movements (RFM) at St Mary's Hospital (Manchester, UK) were approached to participate in the study as previously described (Dutton *et al.* 2012; Warrander *et al.* 2012). Written informed consent was obtained and the study approved by Oldham and Greater Manchester North Research Ethics Committees (REC no. 08/1011/83 and 11/NW/0664). A diagnosis of depression during pregnancy, including any treatment prescribed, was recorded from the participant's medical notes.

### Queen Charlotte's and My Baby and Me study (MBAM) cohorts

For both cohorts, women awaiting an elective Caesarean section (with no known complications of pregnancy) were recruited from Queen Charlotte's maternity hospital, London. Participants in the Queen Charlotte's cohort (*n* = 40) were recruited between 2010 and 2011, while an independent cohort of women participating in the MBAM (*n* = 81) were recruited in 2014. Written informed consent was obtained. The Queen Charlotte's study was approved by the Ethics Committee of Hammersmith and Queen Charlotte's Hospital, London (REC no. 08/H0708/126) and MBAM was approved by the Research Ethics Committee of London (Chelsea) (REC no. 13/LO/1436).

At the time of recruitment, maternal prenatal depressive symptoms were measured using the Edinburgh Postnatal Depression Scale (EPDS). This questionnaire has been validated for use during pregnancy (Cox *et al.* 1996), with total EPDS scores ranging from 0 (low depression) to 30 (high depression). An EPDS score ⩾13 is used to identify women at risk of a depressive disorder (Cox *et al.* 1987).

### Placental dissection

All placentas were collected immediately after delivery and sampled within 1 h (O'Donnell *et al.* 2012; Warrander *et al.* 2012). Villous trophoblast tissue samples were taken from the maternal surface of the placenta, midway between the cord and distal edge. Sampling methods were comparable across all three independent cohorts. Tissue samples were washed in phosphate-buffered saline to remove maternal blood and stored in RNA*later* (Sigma-Aldrich, UK) prior to storage at −80°C.

### Gene expression analysis

Total RNA was extracted from the placental tissue samples from the Manchester and MBAM cohorts using the GenElute Mammalian Total RNA Miniprep Kit (Sigma-Aldrich, UK). RNA quantity and quality (based on the 260:280 absorbance ratio) were assessed using a NanoDrop ND-1000 spectrophotometer. From the Queen Charlotte's cohort, RNA was extracted from placental tissue samples using RNeasy Mini Kits (Qiagen, UK). In addition, for this cohort RNA integrity was examined using a Bioanalyzer 2100 (Agilent, UK), according to the protocol for a RNA 6000 Nano Assay; samples with a RNA integrity number (RIN) value of ⩾5 were considered of sufficient quality for reverse transcription and quantitative polymerase chain reaction (qPCR).

For the Manchester cohort, 5 *µ*g of RNA were reverse transcribed using M-MuLV reverse transcriptase (Promega, UK) with random hexamers, according to the manufacturer's instructions. For both the Queen Charlotte's and MBAM cohorts, 2 *µ*g of RNA were reverse transcribed using the Superscript II first strand complementary DNA (cDNA) synthesis system (Invitrogen, UK), according to the manufacturer's instructions.

Quantitative RT-PCR was performed using a Chromo 4 Four Colour Real Time Detector (MJ Research) in a 20 *µ*l reaction containing 5 *µ*l of cDNA (diluted 1 in 50), 1X Buffer 2 mm MgCl_2_, 2 mm deoxynucleotides (dNTPs), 0.65 units Taq [Fermentas (Thermo), UK], 1 *µ*m of each primer (Sigma-Aldrich, UK) and 0.12X Sybr Green (Invitrogen, UK). All samples were run in triplicate and duplicate plates were performed for MBAM. Conditions for amplification were: (1) 15 min at 94°C; (2) 30 s at 94°C; (3) 30 s at 60°C; (4) 30 s at 72°C; and (5) 30 s 75°C, with steps 2–5 repeated for a total of 40 cycles. Melt Curve was performed from 70 to 94°C, reading every 0.5°C and holding for 2 s.

Primer sequences were as follows: *YWHAZ* forward: TTCTTGATCCCCAATGCTTC and reverse: AGTTAAGGGCCAGACCCAGT; *PEG3* forward: CTCACAACACAATCCAGGAC and reverse: TAGACCTCGACTGGTGCTTG (Feng *et al.* 2008); *PEG10* forward: AAATTGCCTGACATGAAGAGGAGTCTA and reverse:AAGCCTAGTCACCACTTCAAAACACACTAAA (Diplas *et al.* 2009); *PHLDA2* forward: GAGCGCACGGGCAAGTA and reverse: CAGCGGAAGTCGATCTCCTT (Apostolidou *et al.* 2007); *CDKN1C* forward: CCCATCTAGCTTGCAGTCTCTT and reverse: CAGACGGCTCAGGAACCATT (Diplas *et al.* 2009); *hPL* forward: CATGACTCCCAGACCTCCTTC and reverse: TGCGGAGCAGCTCTAGATTG (Dutton *et al.* 2012). *hPL* primers were designed to analyse the expression of CSH1/hPL-A and CSH2/hPL-B, from which the majority of circulating placental lactogens is derived (Newbern & Freemark, 2011). Primer specificity was assessed based on gel electrophoresis product band size and qPCR melt curves.

Gene expression data are presented as the ∆CT (target gene expression relative to the housekeeping gene *YWHAZ*) and as the fold change in expression, calculated using the 2^−∆∆CT^ (Livak & Schmittgen, 2001) where the ∆∆CT is the target gene expression relative to expression in the control group. The housekeeping gene *YWHAZ* has previously been demonstrated to be stably expressed in the human placenta (Meller *et al.* 2005; Murthi *et al.* 2008; Cleal *et al.* 2009, 2010). Furthermore, we found no significant difference in *YWHAZ* expression between control and depressed participants in the Manchester cohort (*p* = 0.76) in an initial pilot study.

### Data analysis

Placental gene expression and maternal EPDS data were normally distributed and parametric statistical tests were used to analyse the data. Placental gene expression data were not significantly associated with any maternal demographics listed in Table 1. The effect of potential confounders (infant birth weight, sex and gestational age) was examined using multiple linear regression analysis. To ease interpretation, ∆CT values representing gene expression values have been inverted [*x*(−1)] such that lower values represent decreasing gene expression. Mediation analysis was carried out in IBM SPSS Statistics Version 20 and PROCESS for SPSS version 2.15 (Hayes, 2013). Bootstrap confidence intervals (CIs) were used to assess statistical significance of the indirect effect (Hayes, 2013).

**Table 1.** Participant characteristics of the Manchester, Queen Charlotte's and MBAM cohorts

	Manchester cohort	Queen Charlotte's cohort	MBAM cohort
Birth outcomes			
Fetal sex, *n* %			
Male	37 (49)	22 (55)	44 (54)
Female	38 (51)	18 (45)	37 (46)
Mean gestational age, weeks (s.d.)/range	40 (1.18)/36–42	39 (0.55)/38–41	39 (0.78)/37–41
Mean birth weight, g (s.d.)/range	3382 (461)/2350–4680	3451 (421)/2640–4700	3384 (415)/2212–4392
Mean placental weight, g (s.d.)/range	604 (131)/354–854	619 (134)/387–932	564 (111)/299–873
Mean Apgar score: 1 min (s.d.)/range	9 (0.97)/6–10	9 (0.48)/8–10	9 (0.68)/5–10
Mean Apgar score: 5 min (s.d.)/range	10 (0.12)/9–10	10 (0.34)/9–10	10 (0.34)/9–10
Mode of delivery, *n* (%)			
Elective Caesarean section	6 (8)	40 (100)	81 (100)
Emergency Caesarean section	9 (12)	0 (0)	0 (0)
Vaginal	39 (52)	0 (0)	0 (0)
Instrumental	21 (28)	0 (0)	0 (0)
Maternal characteristics			
Ethnicity, *n* (%)			
Caucasian	50 (67)	29 (72.5)	47 (58)
African/Afro-Caribbean	7 (9)	2 (5)	6 (7)
Indian/Pakistani/Bangladeshi	13 (17)	1 (2.5)	8 (10)
Middle Eastern	2 (3)	1 (2.5)	4 (5)
Far Eastern	0 (0)	2 (2.5)	3 (4)
Other	3 (4)	2 (5)	10 (12)
Do not wish to say	0 (0)	3 (7.5)	3 (4)
Maternal education, *n* (%)			
Left before GCSE/O level	Not recorded	1 (2.5)	0 (0)
GCSE/O level	Not recorded	3 (7.5)	6 (7)
A levels	Not recorded	6 (15)	6 (7)
Vocational training	Not recorded	5 (12.5)	4 (5)
University degree	Not recorded	16 (40)	42 (52)
Higher degree	Not recorded	9 (22.5)	19 (24)
Do not wish to say	Not recorded	0 (0)	4 (5)
Household income, *n* (%)			
<£18 000 per year	Not recorded	3 (7.5)	9 (11)
£18–25 000 per year	Not recorded	3 (7.5)	3 (4)
£25–43 000 per year	Not recorded	8 (20)	8 (10)
>£43 000 per year	Not recorded	18 (45)	37 (46)
Do not wish to say	Not recorded	8 (20)	24 (29)
Mean age, years (s.d.)/range	29 (5.50)/17–42	35 (4.48)/26–42	34 (3.86)/26–41
Mean maternal BMI, kg/m^2^ (s.d.)/range	25 (4.16)/19–38	24.26 (4.08)/19–38	Not recorded
Mean parity (s.d.)/range	1 (1.13)/0–5	1 (1.45)/0–9	2 (1.80)/0–10
Smoking during pregnancy, *n* (%)			
Yes	11 (15)	3 (7.5)	2 (2)
No	64 (85)	37 (92.5)	79 (98)
Alcohol consumption, *n* (%)			
None	74 (99)	32 (80)	76 (94)
1–5 units/week	1 (1)	8 (20)	5 (6)
Mean maternal EPDS score, total (s.d.)/range	Not assessed	8.9 (5.46)/0–25	7.3 (4.66)/0–18
Clinical depression during pregnancy, *n* (%)^a^			
No	68 (91)	40 (100)	Not recorded
Yes	7 (9)	0 (0)	Not recorded

MBAM, My Baby and Me study; s.d., standard deviation; GCSE, General Certificate of Secondary Education; O level, Ordinary level; A level, Advanced level; BMI, body mass index; EPDS, Edinburgh Postnatal Depression Scale.

a A diagnosis of clinical depression was recorded from participants’ medical notes.

## Results

### Manchester cohort

In an initial pilot study, expression of the imprinted genes *PEG3, PEG10, PHLDA2* and *CDKN1C* was analysed in a cohort of placentas (*n* = 75), which included a subset of women (*n* = 7) who had diagnosed depression during pregnancy. Characteristics of the study participants in the Manchester cohort are shown in Table 1. Participants with diagnosed depression did not differ significantly in terms of maternal demographics from control participants (results not shown).

**Fig. 1.**
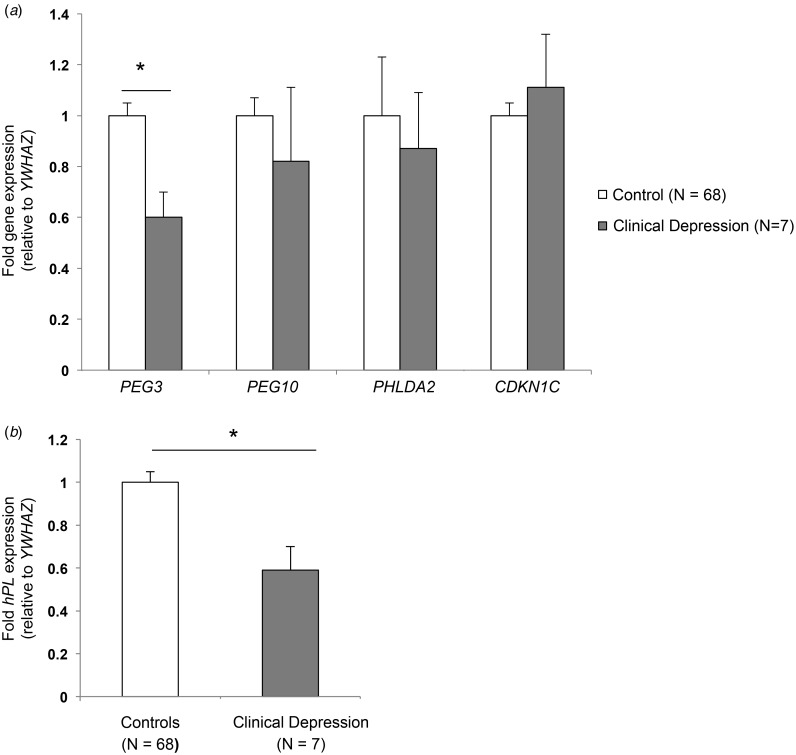
Manchester cohort – maternal diagnosed depression and placental gene expression. (*a*) There was a significant decrease in paternally expressed gene 3 (*PEG3*) expression in women with diagnosed depression during pregnancy. Placental paternally expressed gene 10 (*PEG10*), pleckstrin homology-like domain family a member 2 (*PHLDA2*) and cyclin-dependent kinase inhibitor 1C (*CDKN1C*) expression was not significantly altered. (*b*) Human placental lactogen (*hPL*) expression was also significantly reduced in depressed participants. Values are means of fold gene expression, with standard errors represented by vertical bars. * *p* < 0.05.

There was no significant alteration in the expression of *PEG10, PHLDA2* or *CDKN1C* in association with maternal depression (Fig. 1*a*). However, placental *PEG3* expression was significantly decreased (by 41%) in placentas from depressed participants compared with controls (Fig. 1*a*). The association between placental *PEG3* expression and maternal diagnosed depression remained significant after controlling for infant birth weight, offspring sex and gestational age (*F*_4,73_ = 3.43, *p* = 0.01, *R*^2^ = 0.16), with only maternal depression diagnosis significantly predicting placental *PEG3* expression (*p* = 0.003). Three participants reported prescribed anti-depressant use during pregnancy; the observed decrease in placental *PEG3* expression remained significant with the exclusion of these participants (*p* = 0.02, *n* = 75).

Given the proposed regulation of placental lactogens by *PEG3*, expression of *hPL* was also analysed in this cohort. Placental *PEG3* and *hPL* expression were significantly positively correlated (*r* = 0.26, *p* = 0.03, *n* = 75). There was a significant 44% decrease in placental *hPL* expression in depressed participants compared with controls (Fig. 1*b*).

### Queen Charlotte's cohort

Results from the pilot study indicated a significant decrease in placental *PEG3* and *hPL* expression in a small number of women with clinically diagnosed depression during pregnancy. To determine whether more general symptoms of prenatal depression were similarly associated with aberrant placental gene expression of these two genes, we made use of a second independent cohort (the Queen Charlotte's cohort; *n* = 40) with information on maternal prenatal depression symptoms. The characteristics of the study participants in the Queen Charlotte's cohort are shown in Table 1. Indication for elective Caesarean section (ELCS) included previous section (*n* = 26, 65%), breech presentation (*n* = 7, 18%), maternal request (*n* = 2, 5%) and other (e.g. previous tear, low-lying placenta, *n* = 5, 12%). No participant reported prescribed anti-depressant use during pregnancy.

**Fig. 2.**
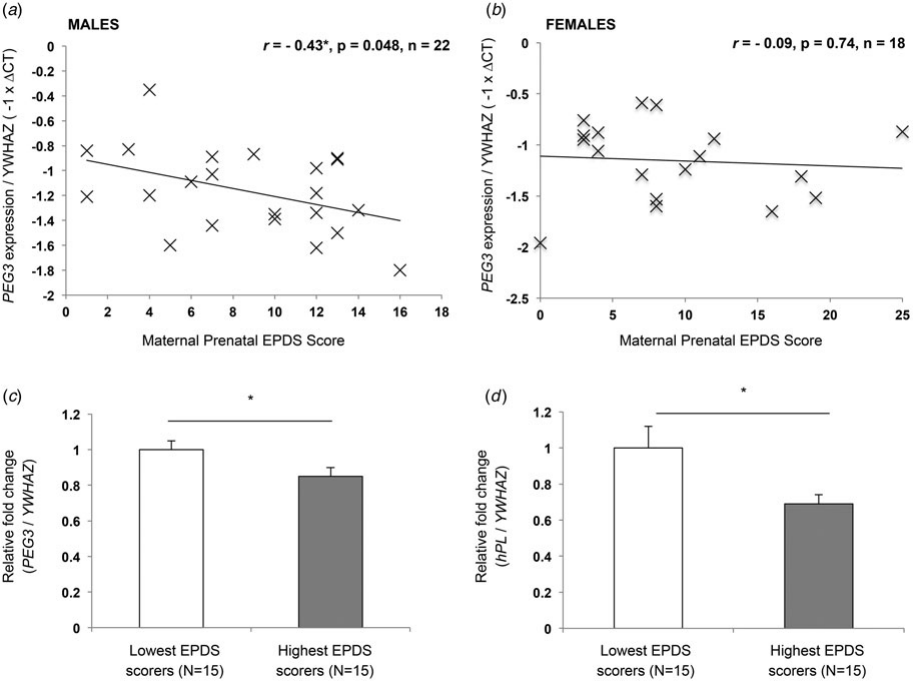
Queen Charlotte's cohort – maternal depression symptoms and placental gene expression. The association between maternal depression and placental paternally expressed gene 3 (*PEG3*) expression appears to be sex specific with a significant inverse association between Edinburgh Postnatal Depression Scale (EPDS) scores and *PEG3* expression in male (*a*) but not female (*b*) placentas. There was a significant decrease in *PEG3* (*c*) and human placental lactogen (*hPL*) (*d*) expression between 15 participants with the lowest and highest EPDS scores. Values are means of fold gene expression, with standard errors represented by vertical bars. * *p* < 0.05.

Despite promising pilot data, the modest correlation between placental *PEG3* expression and maternal prenatal EPDS scores (*r* = −0.23, *p* = 0.16, *n* = 40) failed to reach statistical significance. Placental *PEG3* expression does not differ between male and female placentas from normal, uncomplicated pregnancies (Janssen *et al.* 2015) but male and female human fetuses are known to respond differently to an adverse intra-uterine environment (Clifton, 2010). Therefore, the association between placental *PEG3* expression and maternal depression was also analysed independently in male and female pregnancies. When the *PEG3* expression data were analysed according to fetal sex, this revealed a significant negative association between placental *PEG3* expression and maternal EPDS scores in males (*p* = 0.048) but not females (*p* = 0.74) (Fig. 2*a* and *b*). A 13% decrease in *PEG3* expression was observed in placentas from participants with an EPDS score ⩾13, highlighting them as at risk of a depressive disorder, although this difference did not reach statistical significance (*p* = 0.16) possibly due to the relatively small number of women scoring above this cut-off (*n* = 9). However, when participants in the current study were grouped into lowest (mean EPDS 2.7, *n* = 15) and highest (mean EPDS 14.5, *n* = 15) EPDS scorers there was a significant 15% decrease (*p* = 0.03) in *PEG3* expression in placentas from the highest EPDS scorers compared with the lowest EPDS scorers (Fig. 2*c*).

Placental *hPL* expression was also negatively correlated with maternal EPDS scores (*r* = −0.27, *p* = 0.13, *n* = 40) but without statistical significance in the full cohort or when analysed independently in male (*r* = −0.25, *p* = 0.25, *n* = 22) and female (*r* = −0.29, *p* = 0.26, *n* = 17) placentas. However, as with *PEG3*, a significant 31% decrease (*p* = 0.03) in placental *hPL* expression was observed in the highest EPDS scorers compared with the lowest EPDS scorers (Fig. 2*d*). There was no significant correlation between placental *PEG3* and *hPL* expression (*r* = −0.13, *p* = 0.43, *n* = 40).

### MBAM cohort

We next sought to replicate these findings in the MBAM cohort (*n* = 81), a larger independent cohort of samples with similar information on maternal prenatal depression symptoms. Characteristics of the study participants in the MBAM cohort are shown in Table 1. Indication for ELCS included previous Caesarean section (*n* = 43, 53%), breech presentation (*n* = 15, 19%), maternal request (*n* = 11, 14%), placenta previa (*n* = 6, 7%) and obstetric history (*n* = 6, 7%).

**Fig. 3.**
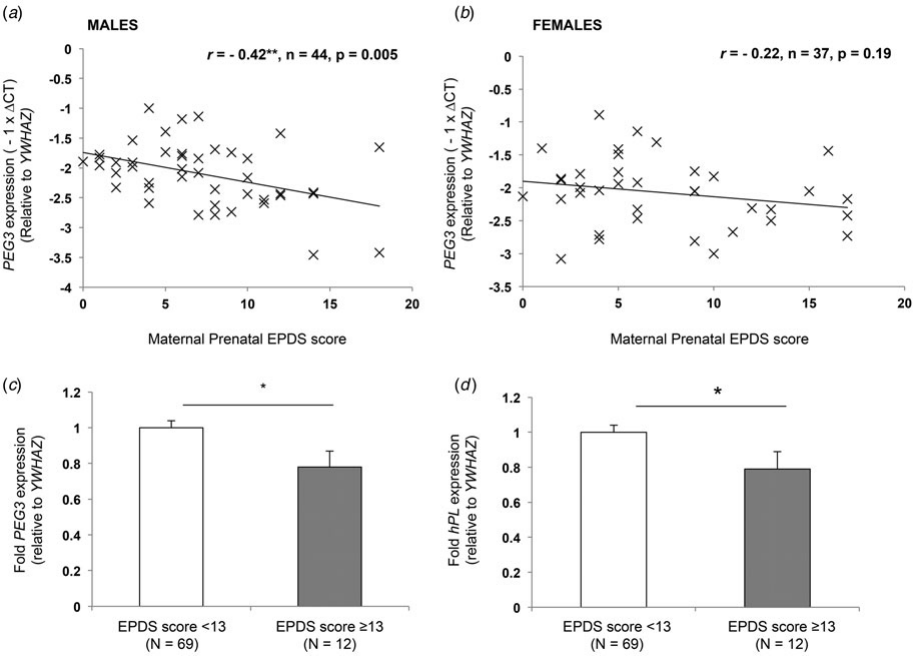
My Baby and Me Study (MBAM) cohort – maternal depression symptoms and placental gene expression. The inverse association between paternally expressed gene 3 (*PEG3*) expression and maternal depression was significant in male (*a*) but not female (*b*) placentas. There was a significant decrease in *PEG3* (*c*) and human placental lactogen (*hPL*) (*d*) expression in participants with Edinburgh Postnatal Depression Scale (EPDS) scores ⩾13 (cut-off for clinical depression). Values are means of fold gene expression, with standard errors represented by vertical bars. * *p* < 0.05.

Placental *PEG3* expression was significantly inversely associated with maternal prenatal EPDS scores (*r* = −0.32, *p* = 0.003, *n* = 81). As with the Queen Charlotte's cohort, when gene expression was analysed according to fetal sex, there was a significant inverse association between *PEG3* expression and maternal EPDS scores in male (*r* = −0.42, *p* = 0.005, *n* = 44) but not female placentas (*r* = −0.22, *p* = 0.19, *n* = 37) (Fig. 3*a* and *b*). Multiple linear regression analysis showed that the association between placental *PEG3* expression and maternal depression symptoms remained significant after controlling for infant birth weight, offspring sex and gestational age (*F*_4,76_ = 2.45, *p* = 0.05, *R*^2^ = 0.11), with only maternal prenatal EPDS scores significantly predicting placental *PEG3* expression (*p* = 0.005). Only two participants reported prescribed anti-depressant use during pregnancy; the association between placental *PEG3* expression and maternal EPDS scores remained significant with the exclusion of these participants (*r* = −0.30, *p* = 0.006, *n* = 79). Finally, a significant 22% decrease in *PEG3* expression was observed in placentas from participants with EPDS scores ⩾13, highlighting them as at risk of a depressive disorder (Fig. 3*c*).

Placental *hPL* expression was also significantly inversely associated with maternal prenatal EPDS scores (*r* = −0.36, *p* = 0.001, *n* = 81) in the overall cohort. As with *PEG3*, this association was sex specific, being significant in male (*r* = −0.51, *p* < 0.001, *n* = 44) but not female (*r* = −0.11, *p* = 0.53, *n* = 37) placentas. There was also a significant 21% decrease in *hPL* expression in placentas from participants with EPDS scores ⩾13 (Fig. 3*d*). Finally, there was a positive correlation between placental *PEG3* and *hPL* expression although this was not statistically significant in the overall cohort (*r* = 0.20, *p* = 0.08, *n* = 81), being significant in male (*r* = 0.39, *p* = 0.01, *n* = 44) but not female placentas (*r* = −0.26, *p* = 0.12, *n* = 37).

### Mediation analysis

Mediation analysis determines the effect of an independent variable on a dependent variable via a third mediating variable and is typically conducted in larger cohort sizes of >100 participants (Fritz & Mackinnon, 2007). In this study mediation analysis was carried out in the two largest cohorts (Manchester and MBAM) with the hypothesis that the association between placental *PEG3* and maternal EPDS scores is mediated by placental *hPL* expression. In the Manchester cohort (*n* = 75) the indirect effect of placental *PEG3* expression on maternal EPDS scores mediated by placental *hPL* expression was not statistically significant in the overall cohort (*B* = 0.29, 95% CI −0.08 to 1.13) or when only male placentas were analysed (*B* = 0.91, 95% CI −0.64 to 5.20). Similarly, in the MBAM cohort (*n* = 81) the indirect effect of placental *PEG3* expression was not statistically significant in the overall cohort (*B* = −0.53, 95% CI −1.71 to 0.46) or when only male placentas were analysed (*B* = 1.35, 95% CI −0.26 to 3.64).

### Maternal depression and birth weight

Maternal stress is known to be associated with an increased risk of fetal growth restriction (Steer *et al.* 1992; Rondo *et al.* 2003; Khashan *et al.* 2008; Liu *et al.* 2012) and therefore we analysed birth weight in relation to maternal depression in the three cohorts. Birth weight was not significantly altered in women with diagnosed depression (3.30 kg *v.* 3.09 kg, *p* = 0.41, *n* = 75) in the Manchester cohort. There was also no significant association between maternal prenatal EPDS scores and birth weight in the Queen Charlotte's (*r* = −0.17, *p* = 0.28, *n* = 40) or MBAM (*r* = 0.12, *p* = 0.28, *n* = 81) cohorts. Reduced expression of *Peg3* in mice has also been linked to fetal growth restriction (Li *et al.* 1999). There was no significant association between placental *PEG3* expression and birth weight in the Manchester (*r* = 0.05, *p* = 0.70, *n* = 75), Queen Charlotte's (*r* = 0.09, *p* = 0.58, *n* = 40) or MBAM (*r* = −0.02, *p* = 0.84, *n* = 81) cohorts. However, it should be noted that for the Queen Charlotte's and MBAM cohorts women with complications of pregnancy (including fetal growth restriction) were excluded and that previous studies demonstrating an association between maternal prenatal stress and fetal growth restriction examined substantially larger cohorts (Talge *et al.* 2007).

## Discussion

This study has identified a novel association between maternal prenatal depression and decreased placental expression of the imprinted gene *PEG3*. In our pilot study, placental expression of the imprinted genes *PEG3, PEG10, PHLDA2* and *CDKN1C* was examined in relation to clinically diagnosed depression. Despite the small number of participants, there was a clear significant reduction in placental *PEG3* expression but no alteration in expression of the other three imprinted genes examined. Importantly, placental *PEG3* expression was also significantly inversely associated with symptoms of maternal prenatal depression in two further independent cohorts, the Queen Charlotte's and MBAM cohorts. This is the first report linking abnormal expression of this imprinted gene in the placenta with maternal depression during pregnancy, which may inform our understanding of the mechanisms underlying the association between prenatal depression and adverse offspring outcomes.

*PEG3* exemplifies all the known functions of imprinted genes; it acts to regulate fetal growth, placental development, behaviour and metabolism in mice (Li *et al.* 1999; Curley *et al.* 2005; Champagne *et al.* 2009; Chiavegatto *et al.* 2012; Kim *et al.* 2013). We did not observe reduced birth weight in this study, probably as a result of the small study numbers and exclusion of growth-restricted infants. However, it is possible that reduced expression of *PEG3* in human pregnancy could contribute to fetal growth restriction, either directly or indirectly via a placental defect, and therefore mediate the previously reported association between maternal prenatal stress and impaired fetal growth.

In this study we also demonstrated a significant inverse association between placental *hPL* expression and maternal prenatal depression in all three cohorts. *hPL* is a lactogenic hormone, produced in large quantities by the placenta, and is important for maternal glucose management during pregnancy and fetal growth (Newbern & Freemark, 2011). Maternal serum hPL levels and placental *hPL* expression have previously been demonstrated to be significantly reduced in pregnancies complicated by fetal growth restriction (Roh *et al.* 2005; Dutton *et al.* 2012). Placental lactogens are closely related to pituitary prolactin (Haig, 2008). This hormone family is known to be important for driving the maternal physiological adaptations to pregnancy (John, 2013) but studies in rodents suggest that these hormones may also be important for psychological adaptation to pregnancy. Both prolactin and placental lactogen can induce maternal behaviour in non-pregnant rodents (Bridges *et al.* 1985, 1990, 1997; Bridges & Freemark, 1995) and prolactin plays an important role in pregnancy-related neurogenesis (Bridges & Grattan, 2003; Shingo *et al.* 2003; Walker *et al.* 2012). There has not yet been a comprehensive examination of serum hPL levels in relation to maternal mood in humans. However, decreased maternal serum levels of prolactin have been reported in human mothers with postnatal depression symptoms (Abou-Saleh *et al.* 1998; Ingram *et al.* 2003; Groer & Morgan, 2007) and increased levels in mothers with low anxiety scores during pregnancy (Asher *et al.* 1995). Prolactin contributes to a suppression of anxiety-related behaviours during pregnancy via binding to prolactin receptors, which are also known to bind the placental lactogens (Torner *et al.* 2001). As with reduced expression of *PEG3*, reduced placental lactogen production could contribute to a suboptimal pregnancy.

*PEG3* has been proposed to regulate the production of placental hormones (John, 2013). We observed a significant correlation between placental *PEG3* and *hPL* expression in the two largest cohorts (Manchester and MBAM). Mediation analysis was carried out in these two cohorts with the hypothesis that the association between placental *PEG3* and maternal depression is mediated by placental *hPL* expression. The indirect effect of placental *PEG3* expression on maternal depression mediated by placental *hPL* expression was not statistically significant in either cohort. However, it should be noted that the sample sizes used in this analysis (Manchester cohort *n* = 75 and MBAM *n* = 81) are relatively low given that mediation analysis is typically conducted in studies of >100 participants (Fritz & Mackinnon, 2007). While it is possible that the gene expression changes observed are independently related to maternal prenatal depression, studies in the mouse placenta demonstrate reduced expression of *Peg3* drives reduced expression of *Prls* (mouse placental lactogens) (Broad & Keverne, 2011; Kim *et al.* 2013). Future studies employing mediation analysis will probably be more informative in larger cohorts and will be crucial in determining the relationship between *PEG3* and *hPL* in the human placenta.

The association between maternal prenatal depression symptoms and placental *PEG3* and *hPL* expression showed a sex bias for both genes, in both cohorts, with the effect more marked with male fetuses. This result is of interest as previous studies suggest sexual dimorphism in infant outcomes following maternal prenatal stress (for a review, see Glover & Hill, 2012). Boys, but not girls, are at an increased risk of attention-deficit/hyperactivity disorder (Li *et al.* 2010), schizophrenia (van Os & Selten, 1998) and impaired motor development (Gerardin *et al.* 2011). There is also some preliminary evidence to suggest that women who give birth to boys are more likely to suffer from postpartum depression than those having girls (de Tychey *et al.* 2008; Lagerberg & Magnusson, 2012). It will therefore be important to establish whether there is an association between placental *PEG3* and *hPL* expression, fetal sex, infant outcomes and maternal postnatal depression.

**Fig. 4.**
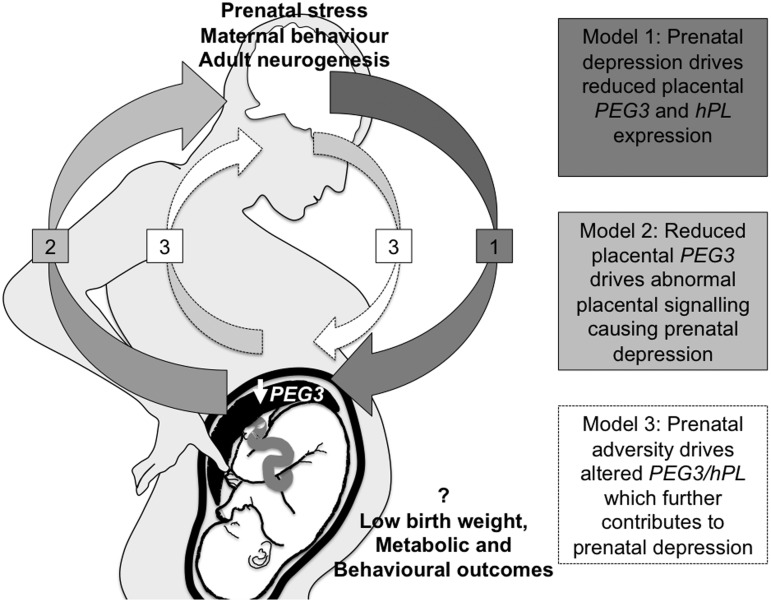
Paternally expressed gene 3 (*PEG3*), human placental lactogen (*hPL*) signalling and maternal psychological adaptation to pregnancy. Model 1: prenatal depression causes reduced *PEG3* and reduced *hPL* expression. Model 2: reduced *PEG3* initiates prenatal depression through regulation of placental lactogen production. Model 3: an adverse intra-uterine environment causes reduced *PEG3* expression limiting placental signalling and establishing a cycle of aberrant placental gene expression, aberrant signalling and abnormal maternal mood.

This study was not designed to establish cause-or-effect relationships. Prenatal depression, or other factors associated with prenatal depression, may drive reduced expression of placental *PEG3* and *hPL* in the placenta (Fig. 4: model 1). *Peg3* in the rodent placenta is known to be responsive to environmental stimuli, such as maternal diet (Broad & Keverne, 2011; Radford *et al.* 2012). Alternatively, reduced *PEG3* may contribute to or even initiate prenatal depression (Fig. 4: model 2). Loss of function of *Peg3* in mice results in abnormal maternal behaviour (Li *et al.* 1999; Champagne *et al.* 2009; Chiavegatto *et al.* 2012). However, in this scenario it is the dams that are mutant for *Peg3* and not the placenta. Rather, aberrant placental *PEG3* expression may contribute to abnormal maternal mood in human pregnancies, via impaired placental production of hPL (Janssen *et al.* 2016). It is also possible that both models are correct. A suboptimal maternal environment, which may include maternal prenatal stress, could misprogramme placental expression of *PEG3*, which in turn may alter placental signalling via *hPL*, thereby further contributing to the depressed mood (Fig. 4: model 3).

There are limitations to this study, primarily in the number of participants. However, this is countered by the reproducibility of these findings in three independent studies. Also, in the Queen Charlotte's and MBAM cohorts, maternal prenatal depression symptoms were assessed 1 day prior to elective Caesarean section when mothers may be particularly anxious. Ideally, future studies would involve assessment of maternal mood at different time points throughout pregnancy in a larger number of participants to provide a better indication of maternal prenatal stress during pregnancy. Another study limitation is that participants in the Manchester cohort suffered from RFM before delivery and so are not representative of uncomplicated pregnancies. However, both controls and women with depression during pregnancy experienced RFM and therefore this was not causative of the differences in gene expression observed. Finally, as depression diagnosis in the Manchester cohort was based solely on a diagnosis reported within clinical records, we cannot exclude the presence of depression symptoms in the control cases. Future studies with measures of depression for all participants (as with the Queen Charlotte's and MBAM cohorts) as well as more detailed information on the nature of depression and medication during pregnancy will be important in confirming the association between aberrant placental gene expression and diagnosed depression.

## Conclusions

In summary, this study is the first to report reduced expression of both *PEG3* and *hPL* in the human placenta in relation to adverse maternal mood. Substantial indirect data from rodent models suggest that this relationship is pathologically relevant and may indeed be causal. Animal studies will be important in further establishing cause-and-effect relationships. Meanwhile, it will be important to validate our findings in a larger study cohort and, importantly, to examine the outcomes of pregnancies with reduced *PEG3* expression, both for the child and for the mother. Measuring maternal serum levels of *hPL* will be instrumental in determining whether reduced placental *hPL* expression in the term placenta reflects reduced hormone serum level during pregnancy. This is of clinical relevance since it may be possible to use maternal serum *hPL* levels as a biomarker in combination with self-report questionnaires to identify mothers at high risk of maternal depression. Finally, our current findings are of broader interest as reduced expression of *PEG3* could provide a mechanistic explanation for the co-occurrence of maternal depression, low birth weight and poorer outcomes for the offspring, a finding that will lead to a greater understanding of both the causes and consequences of prenatal depression.
